# Thrombotic Thrombocytopenic Purpura Following Pfizer-BioNTech COVID-19 Vaccination in a Patient With Multiple Myeloma: Case Report and Literature Review

**DOI:** 10.7759/cureus.46784

**Published:** 2023-10-10

**Authors:** Chitramalya Dan, Akshat Sahai, Deepmalya Dan, Anmol Sahai, Ram Trehan

**Affiliations:** 1 Hematology and Oncology, Greater Washington Oncology Associates, Silver Spring, USA; 2 Cardiology, Texas A&M College of Medicine, Houston, USA; 3 Internal Medicine, College of Medicine and Sagore Dutta Hospital, Kolkata, IND; 4 Internal Medicine, Saraswathi Institute of Medical Sciences, Hapur, IND

**Keywords:** caplacizumab, vitt, ttp, pfizer-biontech vaccine, covid-19 vaccine

## Abstract

The vaccines developed to prevent infection and mitigate morbidity and mortality in patients with COVID-19 demonstrated high efficacy in clinical trials but were associated with adverse events, most of which were mild and transient. However, some adverse events were rather serious, with grave prognoses. Of note, a few cases of autoimmune hematological conditions such as thrombotic thrombocytopenic purpura (TTP), immune thrombocytopenic purpura (ITP), and vaccine-induced immune thrombotic thrombocytopenia (VITT) were reported. TTP following Pfizer-BioNTech mRNA vaccination is exceptionally rare, with very scant literature. This case report describes an interesting case of a 61-year-old woman who presented 22 days after receiving the third dose of the Pfizer-BioNTech mRNA COVID-19 vaccine with malaise, bloody stools, and jaundice. Her medical history was significant for multiple myeloma previously treated with autologous bone marrow transplant and in remission with chemotherapy. She also had a history of chronic heart failure with preserved ejection fraction (HFpEF) and neuropathy treated with daily vitamins. The diagnosis was predicted by her classic presentation and was clinched by low ADAMTS13 activity. She was treated with plasmapheresis, steroids, and monoclonal antibodies. Intriguingly, her hospital stay was further complicated by an episode of generalized tonic-clonic seizure requiring intubation and mechanical ventilation for airway protection. Albeit infrequent, COVID-19 vaccine-associated TTP is associated with substantial morbidity and mortality. Hence, early diagnosis and treatment are essential in patients presenting with thrombocytopenia after COVID-19 vaccination.

## Introduction

The COVID-19 pandemic, caused by the severe acute respiratory syndrome coronavirus 2 (SARS-CoV-2) resulted in millions of deaths around the world. Multiple vaccines (Pfizer-BioNTech, Moderna, Janssen, Novavax) have been developed and have proved to be efficacious and safe. The Pfizer-BioNTech COVID-19 vaccine trials showed efficacy above 90% in preventing COVID-19 and it was authorized for emergency use in December 2022 by US FDA [1]. Being the first marketed vaccine in the US, this mRNA vaccine has an important role in mitigating the COVID-19 pandemic. Since the introduction of vaccines, a plethora of research and case reports have been published regarding the adverse effects of various COVID-19 vaccines. The most common major adverse events reported with the Pfizer-BioNTech vaccine were acute myocardial infarction, myocarditis, pericarditis, appendicitis, and cerebrovascular accident [2].

Thrombotic thrombocytopenic purpura (TTP) is a rare condition attributed to decreased activity levels of ADAMTS13, a disintegrin and metalloproteinase with a thrombospondin type 1 motif, member 13. TTP classically features a pentad of fever, thrombocytopenia, microangiopathic hemolytic anemia, and microvascular thrombosis leading to ischemic organ damage specifically with renal and neurological manifestations [2, 3]. TTP can potentially have a high mortality rate, but survival improves drastically with prompt therapy [3]. Therefore, a high index of suspicion for TTP is recommended in patients who present with thrombotic microangiopathy.

Vaccine-associated TTP is a rare type of acquired TTP that has been reported to develop following the administration of various vaccines including seasonal influenza, pneumococcal, and rabies vaccines, featuring expression of low ADAMTS13 activity levels and high titers of ADAMTS13 antibodies [4, 5]. Initially reported as a consequence of COVID-19 infection, TTP has also been associated with COVID-19 vaccine administration [6, 7]. Vaccines have also been associated with the relapse of TTP in patients in remission [3]. Immune thrombocytopenic purpura (ITP) and vaccine-induced immune thrombotic thrombocytopenia (VITT) are other important differentials that are to be considered in patients presenting with post-vaccination thrombocytopenia [2, 4, 6, 7]. In this case report, we discuss a rare case of acquired TTP, which occurred after the administration of the third dose of the Pfizer-BioNTech mRNA vaccine.

## Case presentation

A 61-year-old African American woman presented to the emergency department (ED) with complaints of malaise, lethargy, dyspnea on exertion, and jaundice, 22 days after receiving the third dose of the Pfizer-BioNTech COVID-19 mRNA vaccine. She started experiencing epigastric pain, nausea, and vomiting one week prior to presentation at the ED, 15 days after vaccination with the third dose of the vaccine. She also reported an episode of blood-streaked stools. She had no complaints of fever, gingival bleeding, epistaxis, altered mentation, dizziness, abdominal pain, diarrhea, or dysuria. She had received doses of the same vaccine prior and had no contraindications to the third dose. Her past medical history was significant for immunoglobin G (IgG) kappa light chain multiple myeloma (MM), heart failure with preserved ejection fraction (HFpEF), and neuropathy. With prior autologous stem cell transplant and carfilzomib therapy, multiple myeloma was in remission with pomalidomide, denosumab, and suppressive acyclovir. She was on torsemide and low-dose aspirin for her cardiovascular condition. There was no past medical or family history of any thrombotic events or autoimmune diseases. At presentation, she was alert, oriented, and cooperative. Her temperature was 36.8^o^C, pulse rate was 84 beats per minute, and blood pressure was 118/66 mm Hg. Her respiratory rate was 23 breaths per minute with oxygen saturation at 92% on ambient air and 98% on 2L/min of oxygen via nasal cannula. Her sclerae were icteric. Bipedal pitting edema was also noted. Systemic examination of the chest, abdomen, and nervous system were otherwise unremarkable. Electrocardiography showed normal sinus rhythm and cardiac troponins were not elevated. COVID-19 polymerase chain reaction (PCR) test showed a negative result. Her hematology and biochemistry profiles (details in Table 1) were significant for normocytic anemia, mild leukocytosis, severe thrombocytopenia, reduced haptoglobin and elevated bilirubin, transaminases, creatinine and lactate dehydrogenase (LDH). She was admitted for further evaluation and was transfused with packed red blood cells (RBCs) and platelets. Aspirin and pomalidomide were stopped. Oral torsemide (10 mg once daily) was continued to prevent volume overload given her history of HFpEF. Based on her presentation and blood reports, she qualified as high risk on the French score and the suspicion for thrombotic thrombocytopenic syndromes, viz., TTP, VITT, and ITP were high on the list of differential diagnoses [3, 8]. She was started on plasmapheresis, methylprednisolone, and caplacizumab. In view of acute kidney injury, a temporary hemodialysis catheter was placed in the right internal jugular vein under sonographic and fluoroscopic guidance with no immediate complications. Her peripheral blood smear demonstrated normocytic anemia with moderate anisopoikilocytosis, numerous schistocytes and microspherocytes, and concomitant marked thrombocytopenia suggesting microangiopathic hemolytic anemia. 

**Table 1:** Lab values at presentation and monitored trends (LDH: lactate dehydrogenase; INR: International Normalised ratio)

Laboratory Test	Normal Values	Day 0	Day 3	Day 5	Day 7	Day 10
Hemoglobin level (g/dL)	12-15	6.9	7.5	7.6	8.0	8.3
Mean Corpuscular Volume (fL)	80-100	90	95	90	96	98.5
Platelet count (/nL)	150–350	8	49	163	278	316
Leukocyte count (/nL)	4.5 - 11	23.13	18.63	10.12	12.76	10.36
Absolute Neutrophil count (/nL)	1.5-7.8	16.56	12.41	9.10	8.3	7.5
Absolute Lymphocyte count (/nL)	1.1–4.8	1.08	3.41	2.38	2.03	2.48
Nucleated RBC count (/nL)	0.00-0.01	2.23	2.74	1.28	0.18	0.00
LDH level (U/L)	135-250	239	-	-	-	200
Haptoglobin level (g/L)	36-195	<10	-	-	-	43
Bilirubin total (g/dL)	<1.2	6	3.9	1.5	0.7	0.6
Aspartate Transaminase (U/L)	<31	92	80	24	11	16
Alanine Transaminase (U/L)	<33	34	84	24	10	18
Alkaline Phosphatase (U/L)	30-130	74	96	15	13	18
Blood Urea Nitrogen	7-25	42	34	-	-	-
Creatinine (mg/dL); NV 0.70–1.20)	0.5-1.2	1.3	1.2	1.0	0.8	0.6
ADAMTS-13 Activity (%)	70-150	-	<5	-	-	-
ADAMTS-13 Antibodies (IU/mL)	<12	-	30	7	2	2
ADAMTS-13 Inhibitor (%)	0-44	-	78.57	-	-	40.38
INR		1.07	1.14	1.30	1.23	1.14
D-Dimer (mg/L FEU)	0.0-0.49	-	>4	-	-	-

Bone marrow aspirate and core biopsy, done to rule out relapse of MM, showed normocellular marrow with trilineage hematopoiesis with relative erythroid and megakaryocytic hyperplasia and no evidence of residual myeloma and negative for lymphoma and acute leukemia. Few scattered plasma cells were present that showed mixed phenotype with no monoclonal plasma cell population on flow cytometry. Luminal fibrin thrombi formation was appreciated in some small vessels. Her blood reports showed significantly reduced ADAMTS13 activity of <5% and high levels of ADAMTS13 inhibitor antibody at 30 U/mL. Her haptoglobin levels were also reduced (Table 1). Test for HIV antibodies, hepatitis screening, and rapid plasma reagin was negative. Levels of antinuclear nuclear antibody, double-stranded DNA (dsDNA) antibodies, antineutrophil cytoplasmic antibodies (ANCA) IgG, rheumatoid factor, and PF-4/HIT (platelet factor-4/heparin-induced thrombocytopenia) antibodies were insignificant. Serum protein electrophoresis was within normal range. Thus, the diagnosis of TTP was confirmed. Transesophageal echocardiography (TEE) demonstrated grade I diastolic dysfunction with left ventricular ejection fraction at 60%. Abdominal ultrasonography (US) showed a normal appearance of the liver with status post cholecystectomy.

She developed a progressively worsening headache and had a single episode of tonic-clonic seizure on the third day of admission that terminated with midazolam, possibly due to severe TTP [2, 4, 5, 6, 8]. Rituximab was added to the treatment regimen. She was also started on levetiracetam for seizure prophylaxis. Her electroencephalogram was negative for further seizure activity. Computed tomography (CT) scan of the head without contrast was negative for any notable findings. On imaging, the chest radiograph was negative for any acute cardiopulmonary disease. CT scan of the chest with contrast showed mild cardiomegaly but no evidence of pulmonary embolus was noted. Doppler ultrasound study of veins of bilateral lower extremities was negative for any femoropopliteal or calf deep vein thrombosis. Her hospital stay was further complicated by a fever on the fifth day, possibly due to aspiration pneumonia and she underwent intubation and mechanical ventilation in the intensive care unit for airway protection. Piperacillin-tazobactam (4.5 g intravenous six-hourly) was started empirically as per local epidemiology and susceptibility patterns and continued for seven days.

**Figure 1:**
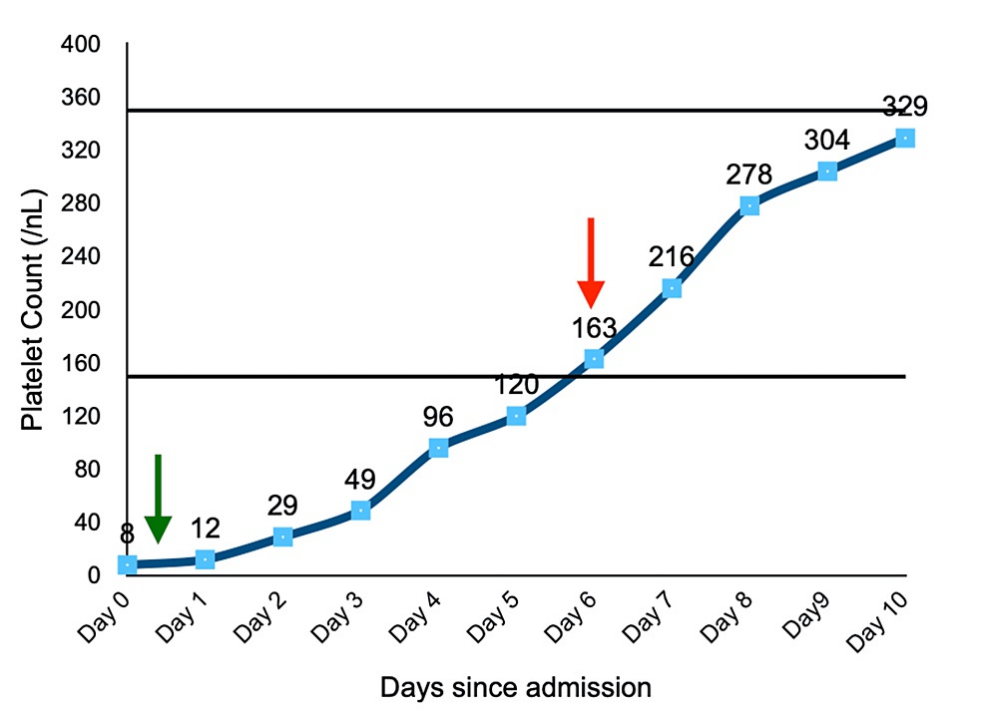
The trend of platelet count during the patient’s hospital course. Normal platelet count range of 150-350/nL is shown. The initiation of plasmapheresis (green arrow) and cessation of plasmapheresis (red arrow) are indicated.

She was extubated on the seventh day as she regained normal levels of consciousness. She had no further episodes of seizures or focal neurological deficits. Plasmapheresis was discontinued once platelet counts moved above 150000/uL (Figure 1). She was discharged on oral 40 mg prednisone with a plan to taper the dose by 10 mg/week and prophylactic sulfamethoxazole-trimethoprim and proton pump inhibitors. She was advised to continue daily caplicizumab (11 mg/day subcutaneous injections) and weekly rituximab (375 mg/m^2 ^infusions) as an outpatient for four weeks. She fared well on her follow-up and her platelet count was found to be within normal range (378/nL) on her most recent outpatient visit, 6 months following the discharge.

## Discussion

TTP is a rare, life-threatening condition affecting three to 10 adults per million population in a year, historically defined by a pentad of fever, thrombocytopenia, microangiopathic hemolytic anemia, and thrombotic events leading to multiple organ-system dysfunctions, specifically of the renal and nervous systems, although this classic pentad is appreciated in less than 10% of patients diagnosed with TTP [2, 3, 8]. TTP ensues secondary to severe reduction of ADAMTS13 activity levels (typically less than 10%), a zinc-containing enzyme that cleaves von Willebrand factor (vWf) multimers into smaller molecules, leading to inadequate degradation of vWF ensuing accumulation of multimers of vWF triggering aggregation and activation of platelets subsequently leading to consumptive thrombocytopenia, thrombosis, and microangiopathic hemolytic anemia. Decreased ADAMTS13 activity may be congenital (5%), due to mutations in the* ADAMTS13 *gene, or acquired (95%), due to autoantibodies against ADAMTS13, often associated with organ transplantation, malignancy, pregnancy, autoimmune diseases, and drugs like clopidogrel, cyclosporine, mitomycin C, quinine amongst others [9]. TTP is predominantly found in females and those of African descent and the median age of presentation is typically around 40 years [9]. Our patient was a 61-year-old African-American female who presented with thrombocytopenia and hemolytic anemia and developed acute kidney injury and neurologic manifestations during her hospital stay, consistent with the classically described demographic and clinical profile.

Definitive diagnosis is based on decreased ADAMTS13 activity levels (< 10% of normal activity), but empiric treatment should be started before ADAMTS13 activity levels are available, based on high clinical suspicion for TTP or increased likelihood of severe ADAMTS13 deficiency based on French or PLASMIC scores [3, 8]. Mortality is 90% without treatment, but survival rates range anywhere from 60% to 90% with prompt treatment [10]. Traditionally treatment is based on plasma exchange and corticosteroids with the inclusion of rituximab and caplacizumab in refractory cases. However, the International Society on Thrombosis and Haemostasis recommends early initiation of caplacizumab [11, 12]. A similar therapeutic strategy was employed for our patient with early initiation of caplacizumab and the addition of rituximab to achieve an adequate response.

The Pfizer-BioNTech vaccine was the first FDA-approved vaccine for the prevention of SARS-CoV-2 and is now authorized for two doses for ages 5 years and over and a third (booster dose) for ages 12 years and older [1, 13]. The most commonly reported side effects include pain at the injection site, myalgia, headache, fever, and nausea due to local or systemic inflammatory responses [14]. The most common major adverse events reported with the Pfizer-BioNTech vaccine were acute myocardial infarction, myocarditis, pericarditis, appendicitis, and cerebrovascular accident [2]. The first case of vaccine-induced TTP was reported in 1960, involving the typhoid vaccine. Since then, other cases of TTP have been reported involving influenza, pneumococcal, H1N1, and rabies vaccines among others [4, 5]. De novo TTP as well as relapse of both congenital and acquired TTP in remission have also been reported with COVID-19 vaccines, with the former variety being more common [3, 9]. We report a de novo case of acquired TTP.

Literature suggests COVID-19 mRNA vaccines can trigger various thrombotic thrombocytopenic syndromes, including TTP, VITT, and ITP through several mechanisms. There is robust evidence of the generation of autoantibodies by the vaccine antigens or the adjuvant components resulting in cross-reactivity with or without aberrant stimulation of the immune system leading to the development of autoimmune diseases [15]. Vaccine-induced formation of immune complexes containing RNA-binding proteins prompts an elevation in serum type-1 interferon immune activity, associated with the production of autoantibodies causing acquired deficiency of ADAMTS13 resulting in TTP [16, 17]. COVID-19 mRNA vaccines have also been shown to induce an acquired deficiency of ADAMTS13 through the increased vWF release from the endothelium or the production of autoantibodies against ADAMTS13 via molecular mimicry between vaccine antigens and ADAMTS13. Occult undiagnosed TTP can often precipitate as full-blown TTP after administration of COVID-19 mRNA vaccines due to activation of platelets and endothelial cells or by triggering the release of vWF multimers. COVID-19 mRNA vaccines can trigger TTP in patients with a history of TTP in remission by providing a ‘second hit’ and aggravating ADAMTS13 deficiency. The causal relationship between acquired TTP and COVID-19 vaccination is primarily supported by temporal correlations rather than by identifying cross-reactive epitopes between antigens in these vaccines and ADAMTS13, an avenue for further research.

Diverse intersecting pathways exist between the thrombotic thrombocytopenic syndromes, specifically TTP and VITT. American Society of Hematology mandates all of the following criteria to diagnose VITT: a history of COVID vaccine administration four to 42 days before symptom onset, any venous or arterial thrombosis (cerebral or abdominal), thrombocytopenia (platelet count less than 150/nL), positive platelet factor 4 heparin-induced thrombocytopenia (PF4-HIT) enzyme-linked immunosorbent assay (ELISA), and markedly elevated D- dimer (> 4 times the upper limit of normal) [7, 18]. In VITT, the presence of PF4 antibodies leads to platelet activation and increased thrombosis in a similar mechanism as heparin-induced thrombocytopenia and is hence treated with non-heparin anticoagulation and intravenous immunoglobulin, as first-line therapy, which is different from the TTP treatment protocol [18]. Due to the high risk of fatal consequences and distinct treatment protocols, TTP and VITT should be distinguished from each other in patients with post-vaccine thrombocytopenia and appropriate therapy should be initiated early. The diagnosis of TTP due to the Pfizer-BioNTech COVID-19 mRNA vaccine was made in our patient after ruling out all the other inciting factors known to secondarily provoke TTP. The reduced levels of ADAMTS13 activity along with high levels of ADAMTS13 antibodies confirmed acquired TTP and the absence of PF4-HIT antibodies ruled out VITT.

## Conclusions

We report a case of TTP following Pfizer-BioNTech COVID-19 mRNA vaccine administration, to raise awareness of this rare albeit serious complication and to highlight the importance of early initiation of therapy. Post-vaccination TTP should be distinguished from VITT, another serious complication of COVID-19 vaccination, by ADAMTS13 activity and antibody levels and PF4-HIT antibody assays, since both are managed differently. Plasma exchange in conjunction with steroids is the mainstay treatment for TTP. Early initiation of caplacizumab and rituximab should be considered with inadequate response. While the benefits of COVID-19 vaccination far outweigh the risks, the possibility of TTP as an adverse effect is to be acknowledged. Further research is needed to evaluate the relationship between COVID-19 vaccines and TTP including comparative epitope profiling of COVID-19 vaccine antigens and ADAMTS-13, and identification of predictive parameters for the development of TTP after COVID-19 vaccination.
